# Characterization of a sesquiterpene synthase and a short-chain dehydrogenase in zerumbone biosynthesis and the applications in engineered *Saccharomyces cerevisiae*

**DOI:** 10.3389/fpls.2025.1635141

**Published:** 2025-10-03

**Authors:** Mengdie Xu, Yimeng Xia, Gaowei Fang, Tangli Li, Jing Ma, Dengyu Li, Qiuhui Wei, Lichan Tu, Xiaopu Yin, Tianyuan Hu

**Affiliations:** ^1^ School of Pharmacy, Hangzhou Normal University, Hangzhou, China; ^2^ School of Pharmaceutical Sciences, Shanghai Jiao Tong University, Shanghai, China; ^3^ Xinchang Pharmaceutical Factory, Zhejiang Medicine Co., Ltd., Shaoxing, Zhejiang, China; ^4^ Department of Pharmacy, School of Medicine, Hangzhou City University, Hangzhou, Zhejiang, China; ^5^ Key Laboratory of Elemene Class Anti-Cancer Chinese Medicines; Engineering Laboratory of Development and Application of Traditional Chinese Medicines; Collaborative Innovation Center of Traditional Chinese Medicines of Zhejiang Province, Hangzhou Normal University, Hangzhou, China; ^6^ State Key Laboratory for Quality Ensurance and Sustainable Use of Dao-di Herbs, China Academy of Chinese Medical Sciences, Beijing, China

**Keywords:** zerumbone, α-humulene, sesquiterpene synthase, short-chain dehydrogenase, *Curcuma wenyujin*

## Abstract

**Introduction:**

Zerumbone is a pharmacologically active sesquiterpenoid with limited availability. This study aims to elucidate its biosynthetic pathway in *Curcuma wenyujin* by identifying and characterizing the key enzymes responsible for its production.

**Methods:**

Candidate genes were selected via transcriptome analysis and phylogenetics. CwTPS8 and CwSDR1 were cloned and functionally characterized using in vitro enzyme assays and heterologous expression in engineered *Saccharomyces cerevisiae*. Molecular docking and site-directed mutagenesis were applied to investigate the catalytic mechanism of CwTPS8.

**Results:**

CwTPS8 was identified as a multifunctional sesquiterpene synthase that catalyzes the formation of α-humulene (a key zerumbone precursor) and β-caryophyllene as main products, along with several minor sesquiterpenes. Mutagenesis studies identified critical residues (e.g., Thr437, Cys436) that significantly shift product specificity toward α-humulene. CwSDR1 was characterized as a short-chain dehydrogenase that efficiently oxidizes 8-hydroxy-α-humulene to zerumbone. A de novo biosynthetic pathway was reconstructed in yeast, resulting in zerumbone production at 0.50 μg/L.

**Discussion:**

This study expands the genetic toolkit for zerumbone biosynthesis and provides insights into enzyme engineering and metabolic engineering strategies to enhance production. Limitations in precursor supply and catalytic efficiency highlight areas for future optimization.

## 1 Introduction

**Figure 1:**
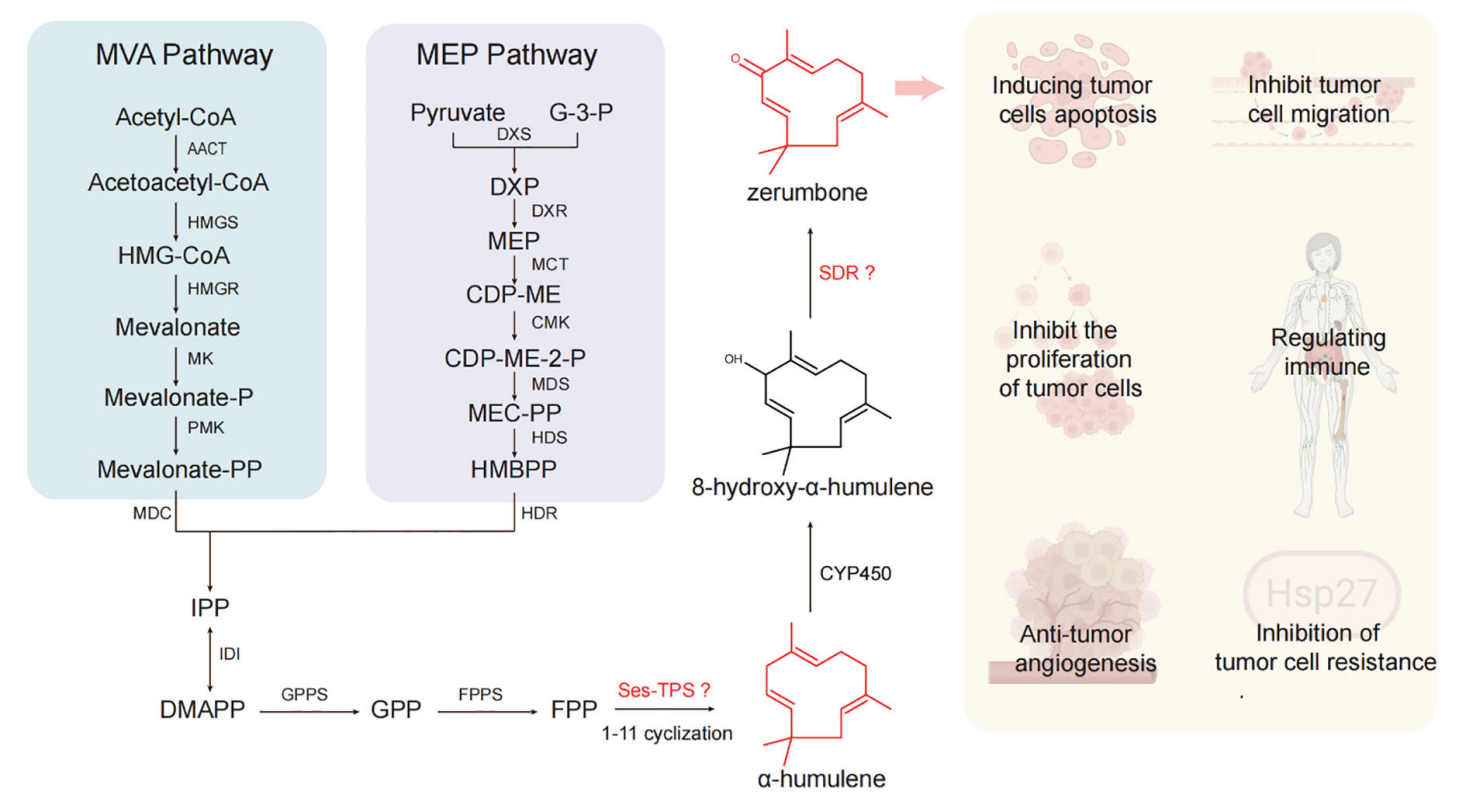
The biosynthetic pathway of zerumbone and its pharmacological effects. Illustrations used in pharmacological effects of zerumbone created with biorender.

Zerumbone, an important humulene-type sesquiterpenoids, exhibits antitumor effects through multiple mechanisms: inhibiting tumor cell growth and proliferation (Urla et al., 2023), inducing tumor cell apoptosis (Li et al., 2023; Wang et al., 2023), suppressing tumor cell migration and invasion (Memari et al., 2022), inhibiting tumor angiogenesis (Tsuboi et al., 2014), modulating immune function (Li et al., 2020; Alsaffar et al., 2023), and reversing tumor multidrug resistance (Choi et al., 2011) (**Figure 1**). However, the sustainable supply of zerumbone is constrained by limited plant-derived raw materials and the challenges of chemical synthesis due to its complex stereochemistry (Kumar et al., 2025). In recent years, advances in biotechnology have positioned biosynthetic approaches as an alternative for the sustainable production of many natural products. Notably, the complete biosynthetic pathway of zerumbone has been elucidated, which includes an α-humulene synthase gene, a cytochrome P450 (CYP450) gene, and a short-chain dehydrogenase/reductase (SDR) gene (**Figure 1**) (Yu et al., 2008; Okamoto et al., 2011; Yu et al., 2011). Despite the successful characterization of the zerumbone biosynthetic pathway, the limited number of characterized genes and their suboptimal catalytic efficiencies present a significant bottleneck, severely restricting heterologous production titers in microbial cell factories.

Sesquiterpenoids exhibit broad applications in pharmaceutical, food, and cosmetic industries (Mai et al., 2021). Previous studies have isolated various sesquiterpenoid types from *C. wenyujin*, including cadinane-type and germacrane-type compounds (Chu et al., 2019; Lin et al., 2019; Li et al., 2022; Liu et al., 2023; Wu et al., 2025). Their carbon skeletons are formed through farnesyl pyrophosphate (FPP) cyclization catalyzed by sesquiterpene synthases, where FPP is synthesized via condensation of isopentenyl pyrophosphate (IPP) and dimethylallyl pyrophosphate (DMAPP) in either the mevalonate (MVA) or methylerythritol phosphate (MEP) pathways (Vattekkatte et al., 2018; Durairaj et al., 2019; Ma et al., 2025). As the key enzymatic step in sesquiterpenoid biosynthesis, sesquiterpene synthase family members contain conserved metal-binding motifs: the “DDxxD” motif and “(N/D)Dxx(S/T)xxxE” motif (Christianson, 2006). These enzymes are classified into acyclic and 1-6, 1-7, 1-10, 1–11 cyclization patterns based on their FPP cyclization modes (Segura et al., 2003; Nie et al., 2024). Notably, humulene synthase—which catalyzes FPP conversion into the zerumbone precursor—belongs to the 1–11 cyclization- type sesquiterpene synthases.

The short-chain dehydrogenase/reductase (SDR) superfamily, one of the three ancient oxidoreductase superfamilies, is characterized by a length of 250–350 amino acids and conserved structural features, including a Rossmann fold that binds to the NADH or NADPH, as well as a coenzyme-binding motif (TGxxxGxG) and a catalytic motif (YxxxK) (Labesse et al., 1994; Kramm et al., 2012; Chen et al., 2019; Marmont et al., 2020). A systematic classification based on conserved domains and catalytic functions divides SDRs into seven evolutionary subtypes: classical, extended, intermediate, complex, divergent, atypical, and unknown. These subtypes regulate plant secondary metabolism through NAD(P)H-dependent mechanisms (de Kraker et al., 2001; Kavanagh et al., 2008). The redox reactions mediated by the Rossmann fold are particularly crucial in terpenoid and alkaloid metabolism, as exemplified by peppermint SDRs that catalyze the stereoselective oxidation of (-)-trans-isopiperitenol (Davis et al., 2005; Fellows et al., 2018), and the SDR in opium poppy catalyzes the dehydrogenation of narcotine hemiacetal to noscapine (Chen and Facchini, 2014). SDRs also play a role in modulating lipid metabolism and stress-response pathways: In *Elaeis guineensis*, SDR suppresses saturated fatty acid biosynthesis and enhances their catabolism (Zhang et al., 2023a), *Oryza sativa* SDRs activate phytoalexin biosynthesis under stress (Kitaoka et al., 2016), and cinnamyl alcohol dehydrogenase modulates polyphenol biosynthesis, thereby enhancing drought tolerance (Wang et al., 2017). This functional versatility establishes SDRs as essential tools for redox regulation and stress-resistance engineering (Stavrinides et al., 2018).

To enrich the genetic resources of the zerumbone biosynthetic pathway and establish efficient microbial cell factories, this study employed transcriptomic data mining and functional characterization to successfully identify two key enzymes: *CwTPS8*, which exhibits humulene/caryophyllene synthase activity, and short-chain dehydrogenase *CwSDR1*, which specifically catalyzes the oxidation of 8-hydroxy-α-humulene to zerumbone. Furthermore, the catalytic mechanism of enzyme *Cw*TPS8 was elucidated, and a *Saccharomyces cerevisiae* cell factory was constructed for the heterologous biosynthesis of zerumbone. These findings provide essential theoretical groundwork for the subsequent development of high-yield yeast chassis for zerumbone bioproduction.

## 2 Materials and methods

### 2.1 Treatment of *Curcuma wenyujin*

The *C. wenyujin* used in this study was harvested from the Rui’an area of Wenzhou, Zhejiang Province, and was confirmed to be a genuine herb by Prof. Guo Zengxi of the Zhejiang Provincial Food and Drug Inspection Institute. Explants were taken from the junction of the root and stem, approximately 2 cm in length, and were inoculated onto modified MS solid medium containing 3 mg/L of 6-benzylaminopurine after surface disinfection. The explants were then cultured in an artificial climatic chamber at 22°C with a 12-hour light/12-hour dark photoperiod to induce clumped shoot formation. After a 30-day period, the clumped shoots were separated into individual plants and transferred to MS medium for continued culture until they reached the three-leaf stage. At the experimental treatment stage, uniform seedlings from a single group were selected and placed in MS liquid medium containing 250 μM methyl jasmonate (MeJA) for 1 hour and 6 hours, respectively. MS liquid medium without MeJA served as the blank control group. All treatments were conducted in a constant temperature light incubator set at 22°C with a photoperiod of 12 hours light and 12 hours dark. Three biological replicates were established for each treatment group. At the conclusion of the treatments, plant leaf samples were rapidly collected and transferred to an ultra-low-temperature freezer at -80°C for storage, following a quick-freezing treatment in liquid nitrogen. This process ensured the provision of standardized biological samples for subsequent RNA extraction experiments.

### 2.2 Bioinformatics analysis

Based on transcriptome data analysis, a total of 3 terpene synthase genes and 3 dehydrogenase genes were identified from *C. wenyujin*. These genes were compared with several currently reported terpene synthase and short-chain dehydrogenases using neighbor-joining (NJ) phylogenetic analysis conducted with MEGA 7. In the MEGA 7 software, using a method with 1,000 bootstrap replicates, one candidate α-humulene synthase (*Cw*TPS8) and one short-chain dehydrogenases (*Cw*SDR1) were identified.

### 2.3 RNA isolation and gene cloning

RNA was extracted from the methyl jasmonate-treated warm tulip samples (flowers, leaves, and stems) using the Total RNA Fast Extraction Kit FastPure Plant (RC401, Vazyme, Nanjing, China). One microgram of RNA was reverse-transcribed into complementary DNA (cDNA) using the Reverse Transcription Kit (RR092A, TaKaRa Japan), and the resulting cDNA was stored at -20°C. Primers were designed based on transcriptome data using Primer Premier 5.0 software (**Supplementary Table S1**). The complete open reading frames (ORFs) of *CwTPS8* (genebank: PV682578) and *CwSDR1* (genebank: PV682579) were obtained through amplification with 2× Phanta Max Master DNA polymerase (Nanjing Vazyme), using the cDNAs as templates. The amplified target fragments were then ligated into the B-Zero-Blunt vector and transformed into competent *E. coli* cells. After incubation, single colonies were selected for PCR with primers, and the clones with the correct bands were sequenced by Tsingke Biotech (Beijing, China). The successfully cloned strains were stored at -80°C.

### 2.4 Conserved structural domains and physicochemical property analysis

Amino acid multiple sequence comparisons of *Cw*TPS8, *Os*TPS3, *Zp*TPS1, *Zp*TPS6 and ZSS1, as well as *Cw*SDR1, *At*SDR1, *Li*BDH and *So*BDH2 were conducted using DNAMAN software. The analysis revealed that *Cw*TPS8 contains the conserved motifs characteristic of terpene synthases, including “RxR”, “DDxxD” and “(N/D)Dxx(S/T)xxxE”; *Cw*SDR1 possess the coenzyme-binding domain “TGxxxGxG” and the catalytic triad “SYK”. These domains were further analyzed for their physicochemical properties using tools available at (https://web.expasy.org/protparam/).

### 2.5 Plasmid construction

The target fragments were amplified using PCR with 2× Phanta Max Master DNA polymerase. The vectors pESC-Trp, pESC-Ura, pESC-Leu and pMAL-His (plasmid pMAL-c2x with His(8)-tag) were linearized with restriction endonucleases at the BamHI or EcoRI sites. The purified target fragments and linearized vectors were combined with Assembly Mix according to the instructions of the pEASY^®^-Uni Seamless Cloning and Assembly Kit (TransGen Biotech) and incubated at 50°C for 30 minutes. The resulting constructs were then transformed into the Trans T1 competent cells and plated on Luria-Bertani (LB) agar plates containing 100 μg/mL ampicillin. Successfully sequenced monoclonal colonies were subsequently selected for storage at -80°C. The primers for the seamless clones are shown in **Supplementary Table S1**.

### 2.6 Protein purification and *in vitro* enzymatic reaction of *Cw*TPS8

Recombinant plasmid pMAL-His::*Cw*TPS8 and the control plasmid pMAL-His were transformed into *E. coli* strain BL21 (DE3) and plated on LB solid medium containing 100 mg/mL ampicillin. Positive clones were selected and inoculated into LB medium containing ampicillin for 20 hours of shaking culture at 37°C and 200 rpm. The following day, the clones were inoculated 1:100 into 100 mL of LB medium containing the corresponding antibiotic and incubated at 37°C and 250 rpm until the optical density at 600 nm (OD_600_) reached 0.6-0.8. IPTG was then added to a final concentration of 0.4 mM, and the cultures were induced by incubation at 25°C and 180 rpm for 18–20 hours. At the end of the induction, the cells were harvested using a cryo-centrifuge at 4°C, 7000 g for 10 minutes, and the medium was poured off to collect the organisms. Add 2 mL of enzyme buffer (40 mM Tris-HCl, pH 8.0; 20 mM imidazole; 250 mM NaCl) to resuspend the cells, then place them on ice. Break the cells using an ultrasonic crusher (30% power, ultrasound for 3 seconds at 10-second intervals for 5 minutes). Centrifuge at 4°C, 9000 × g for 45 minutes, collect the supernatant proteins, and combine them with an appropriate amount of Ni^2+^ at 4°C for 2–3 hours to facilitate binding. The column was pre-equilibrated with a buffer solution (20 mM Tris-HCl, pH 8.0; 250 mM NaCl). The supernatant proteins were then loaded onto the nickel-nitrilotriacetic acid (Ni-NTA) column using a pre-cooled buffer (20 mM Tris-HCl, pH 8.0; 30 mM imidazole; 250 mM NaCl) to wash away the heterogeneous proteins. Subsequently, a buffer containing (20 mM Tris-HCl, pH 8.0; 100 mM imidazole; 250 mM NaCl) was used to elute the target proteins. Finally, the target proteins were concentrated using ultrafiltration tubes at 4°C and 4000 rpm. They were washed three times with ultrapure water for 30 minutes each time and then exchanged twice with cryoenzyme buffer (50 mM HEPES, 10 mM MgCl_2_, 5 mM DTT, 5% (v/v) glycerol, pH 7.5) before partitioning. The electrophoretic analysis of the purified protein is presented in **Supplementary Figure S1**. The purified proteins were flash-frozen in liquid nitrogen immediately after purification and maintained at −80°C for storage. Protein concentration was quantified using the BCA Protein Assay Kit, employing bovine serum albumin (BSA) as a calibration standard. Enzymatic reactions were conducted by incubating the purified proteins with FPP substrate at 30°C with 120 rpm shaking for 3 hours, with the reaction mixture overlaid with an equal volume of *n-*hexane. Following the reaction, the hexane phase was carefully separated, concentrated to 200 µL under a gentle nitrogen stream, and preserved at 4°C.

To determine the catalytic activity of the recombinant protein *Cw*TPS8, it was diluted to a concentration of 20 µg/mL using cryoenzyme buffer (50 mM HEPES, 10 mM MgCl_2_, 5 mM DTT, 5% (v/v) glycerol, pH 7.5). Various concentrations of FPP (10 µM, 25 µM, 50 µM, 100 µM, 125 µM, and 150 µM) were prepared with an equal volume of *n-*hexane and incubated for 10 minutes at 30°C. EDTA was then added to the reaction mixture to halt the reaction, followed by thorough mixing using vortexing. The mixture was centrifuged at 10,000 *g* for 1 minute, and the *n-*hexane layer was collected. After extracting with *n-*hexane three times, the extracts were concentrated to 70 µL under a stream of nitrogen for GC-MS detection. The *K*_m_ and *V*_max_ values were calculated using GraphPad Prism. All data were derived from three independent experiments.

### 2.7 Functional characterization of *Cw*TPS8 and *Cw*SDR1 in *S. cerevisiae*

The recombinant plasmid pESC-Trp::*Cw*TPS8 as well as the control plasmid pESC-Trp were transformed into yeast cell (using TransGen Biotech’s Frozen Yeast Transformation II™ Kit). The cells were then cultured on solid SD-Trp medium for 3 days at 30°C. Positive clones were selected and incubated in SD-Trp medium for 20 hours. After this period, the OD_600_ was diluted to 0.2, and after an additional 12 hours, it was further diluted to 0.05 to initiate fermentation using 100 mL of SD-Trp medium containing glucose at 30°C and 200 rpm to start fermentation. After two days, the medium was replaced with 100 mL of defective medium containing galactose for an additional three days to induce gene expression. At the end of fermentation, the fermentation products were extracted through ultrasonic crushing using an equal volume of *n-*hexane, repeated three times, and concentrated using a rotary evaporator at 42°C and 70 rpm. The products were then dissolved in 1 mL of *n-*hexane, filtered through a 0.22 μm organic membrane, and stored at 4°C.

### 2.8 The construction of a yeast strain for the *de novo* synthesis of zerumbone

In a previous study, we developed an engineered yeast strain, designated FY94, capable of producing high levels of FPP (Hu et al., 2024). In this strain, the key genes involved in the FPP biosynthetic pathway—*tHMG1*, *IDI*, and *ERG20*, were overexpressed, while the coding gene for Erg9, which catalyzes the conversion of FPP to squalene, was downregulated. Additionally, three genes, *ROX1*, *YJL064w* and *YPL062w*, that reduce the metabolic flux of MVA pathway were knocked out. Here, the recombinant plasmid pESC-Trp::ZSS1 was transformed into strain FY94, resulting in the new strain FY94-1. Next, the plasmid pESC-Leu::CYP71BA1-*At*CPR was introduced into FY94–1 to produce 8-hydroxy-α-humulene, leading to the creation of strain FY94-2. Finally, the plasmid pESC-Ura::*Cw*SDR1 was transformed into FY94–2 to produce zerumbone, resulting in the final strain, FY94-3. The strains used in this study are listed in **Supplementary Table S2**.

### 2.9 Molecular docking and targeted mutation experiments

The protein sequence of *Cw*TPS8 was submitted to SWISS-MODEL (http://swissmodel.expasy.org), using the structure of alpha-humulene synthase (B1B1U3.1.A) as a template to generate the protein structure model of *Cw*TPS8. After obtaining the 3D structure of the substrate FPP using ChewBio3D, molecular docking of *Cw*TPS8 and FPP was conducted in AutoDock Vina and subsequently visualized and analyzed structurally using PyMOL.

Using pESC-Trp::*Cw*TPS8 as a template, the key amino acids of *Cw*TPS8 were targeted for mutagenesis with the QuickChange Site-Directed Mutagenesis Kit (TransGene Biotech, Beijing, China) (see **Supplementary Table S1** for mutation primers). The mutated vector was transformed into the *Saccharomyces cerevisiae* FY95 sensory strain (FY95 is an engineered yeast that produces more FPP than FY94) for qualitative and quantitative analysis and the genotype of FY95 was showed in **Supplementary Table S2**. The transformation and the first two activation steps are described in section 2.7. The key difference from the method outlined in section 2.7 is the addition of 10% *n-*dodecane to cover the fermentation after the transition to galactose medium. Bidirectional extraction was performed, and the *n-*dodecane layer was collected at the end of fermentation. This *n-*dodecane layer was then diluted 50-fold with *n-*hexane for product characterization and quantitative analysis using GC-MS. The samples were stored at 4°C.

### 2.10 GC-MS analysis

The qualitative and quantitative analyses of the samples were conducted using an Agilent 8890-7000GC/TQ gas chromatography-tandem triple quadrupole mass spectrometer (Agilent, CA, USA) system under the following analytical conditions: A DB-5ms capillary column (30 m × 0.25 mm × 0.25 μm) was utilized with an injection volume of 1 μL. The temperature program commenced at 80°C, followed by a gradient increase of 10°C·min^-1^ to 240°C (held for 2 minutes), and then ramped at 20°C min^-1^ to 260°C (held for an additional 2 minutes). Instrument parameters included an electron energy of 70 eV, an injector temperature maintained at 275°C, and an ion source temperature set at 280°C. Full scan mode was employed across a mass-to-charge (m/z) range of 30–350 for comprehensive spectral acquisition. β-Caryophyllene (purity ≥95%) and cryptomeridiol (purity ≥95%) standards were obtained from Aladdin Biochemical Technology Co., Ltd. (Shanghai, China). Zerumbone (purity ≥95%) was acquired from Sigma-Aldrich. The synthetic gene fragment encoding α-humulene synthase (GenBank Accession No. AB247331.1), used as a positive control, was supplied by Genecefe Biotechnology Co., Ltd. (Jiangsu, China). β-Caryophyllene was used to generate a calibration curve for quantifying both α-humulene and β-caryophyllene, as their structural similarity and physicochemical properties are expected to yield comparable instrumental response factors. The standard curve of β-caryophyllene is provided in **Supplementary Figure S2**.

## 3 Results

### 3.1 The screening and cloning of candidate humulene synthase gene

**Figure 2:**
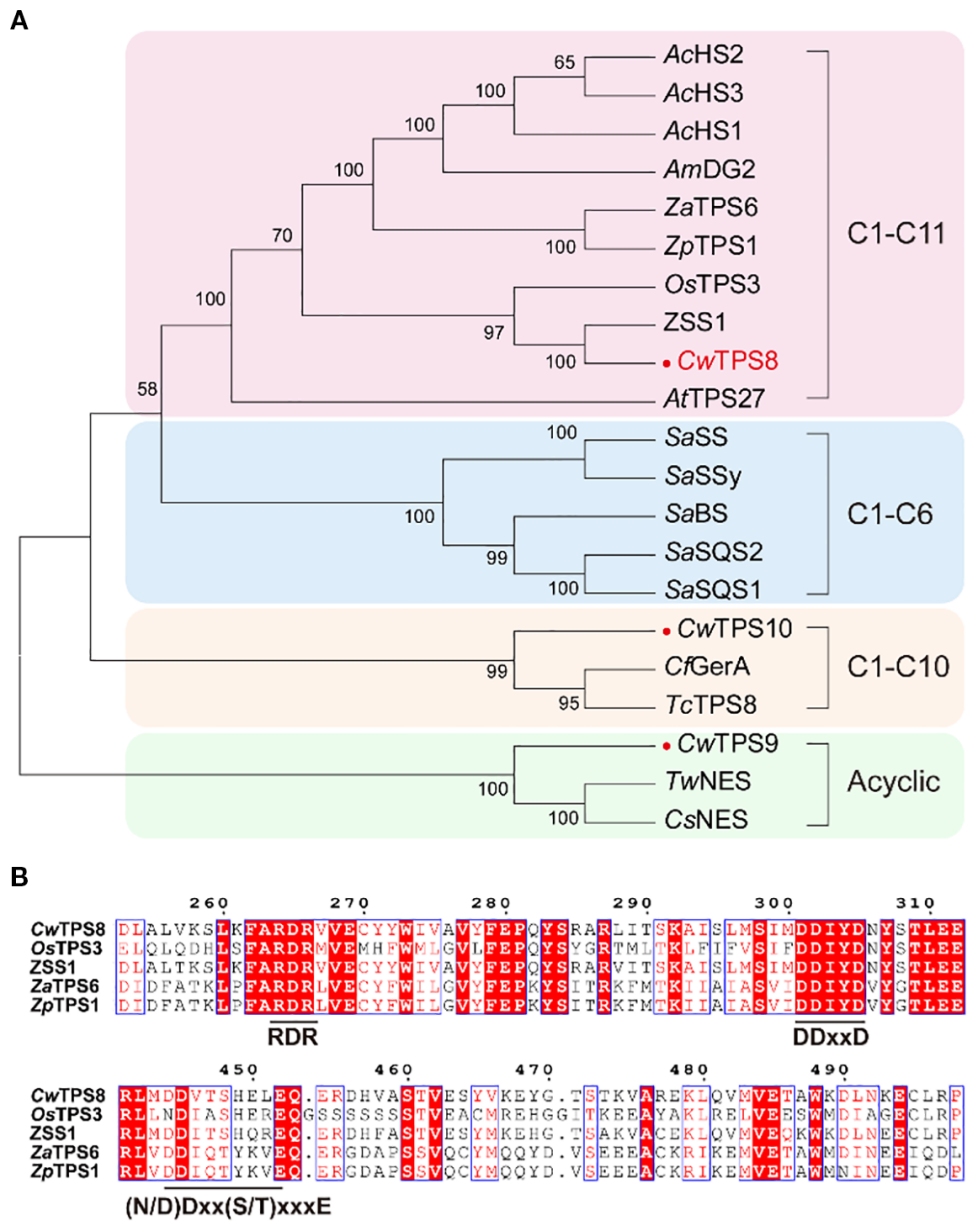
The phylogenetic analysis and conserved motif analysis of *CwTPSs*. **(A)** The phylogenetic tree of *CwTPS*s. The genes used in this analysis are listed in **Supplementary Table S3**; **(B)** The conserved motif analysis of *CwTPS8* which has a conserved basic residue cluster “RxR” motif, the metal-binding “DDxxD” motif and the substrate-stabilizing “(N/D) Dxx(S/T) xxxE” motif.

In previous study, we performed transcriptome sequencing on *C. wenyujin* (Wei et al., 2022). Based on the gene annotation, three candidate sesquiterpene synthase genes were screened. To predict the functions of these genes, phylogenetic analysis was conducted (**Figure 2A**). The result showed that one of the candidate genes, named *CwTPS8*, was clustered into a clade with sesquiterpene synthase genes that catalyze the 1,11-cyclization of the substrate FPP, and exhibited close phylogenetic proximity to a functionally characterized humulene synthase gene, *ZSS1*. Whereas, *CwTPS9* and *CwTPS10* might produce acyclic sesquiterpene and products with 1,10-cyclization, respectively. The multiple alignments of *CwTPS8* and humulene synthase genes from other plants showed that *CwTPS8* has the metal-binding “DDxxD” motif and the substrate-stabilizing “(N/D) Dxx(S/T) xxxE” motif which were conserved in the sequences of class I terpene synthases (**Figure 2B**). Besides, a conserved basic residue cluster facilitating carbocation cascade termination in cyclic sesquiterpene biosynthesis, “RxR”, was also found in *CwTPS8* (**Figure 2B**). Integrating phylogenetic and conserved domain analyses, we propose TPS8 encodes a 1,11-cyclizition sesquiterpene synthase with specific humulene-producing activity.

### 3.2 *Cw*TPS8 is a β-caryophyllene/α-humulene synthase

To determine whether *Cw*TPS8 functions as the key sesquiterpene synthase in the zerumbone biosynthetic pathway, we characterized its activity through both *in vitro* enzymatic reactions and *in vivo* yeast fermentation assays.

**Figure 3:**
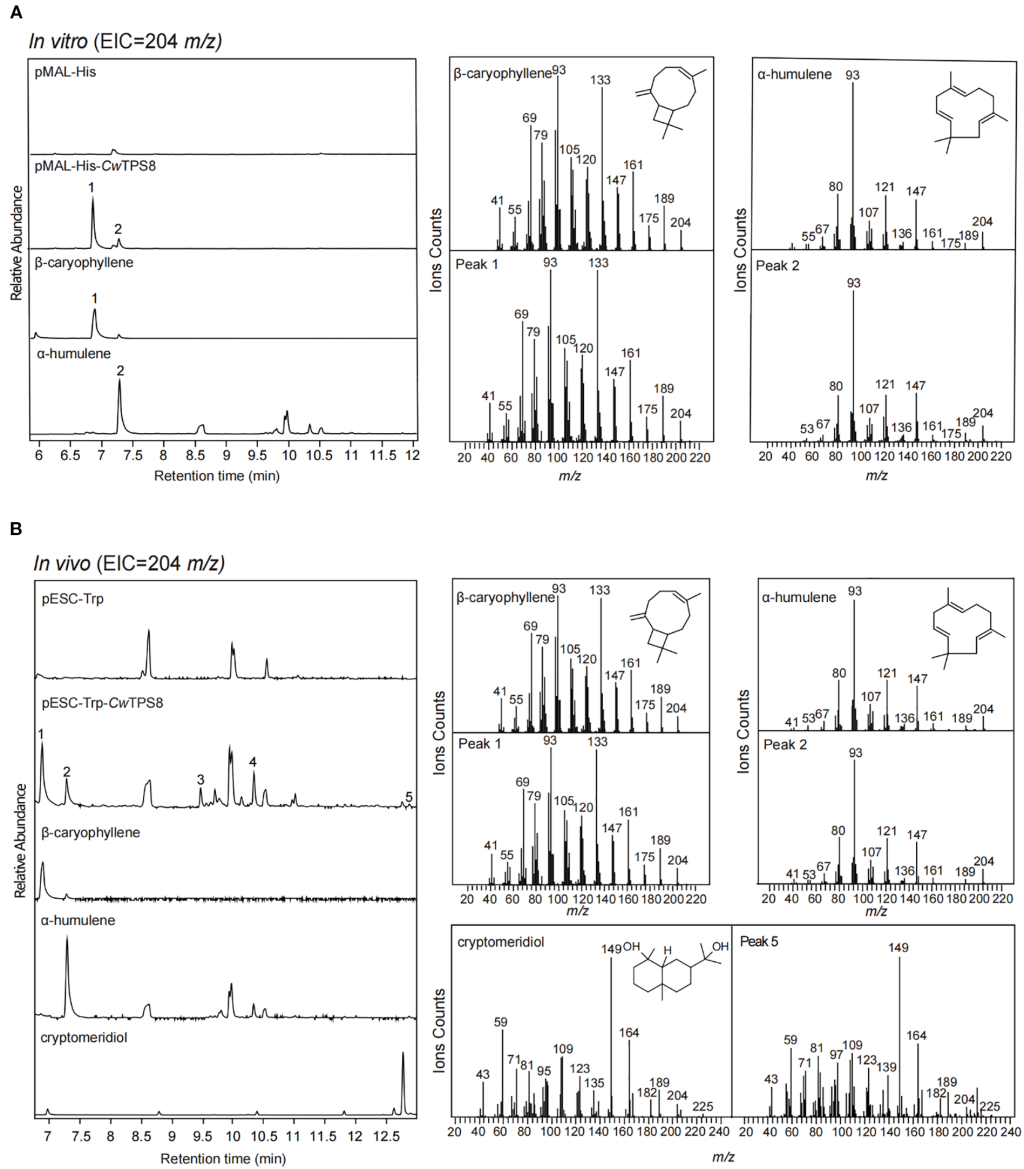
GC-MS analysis of the products of *Cw*TPS8. **(A)** GC-MS analysis of the products of the *in vitro* enzymatic reaction of *Cw*TPS8. Standards of β-caryophyllene, α-Humulene is a product of enzyme with characteristic function. **(B)** GC-MS analysis of the products of yeast strains expressing *Cw*TPS8. Standards of cryptomeridiol (95% purity).

Firstly, *CwTPS8* was cloned into the prokaryotic expression plasmid pMAL-His and subsequently transformed into *E. coli* BL21 (DE3). The recombinant proteins were induced using IPTG. After extraction and purification, the recombinant proteins were incubated with the substrate FPP. GC-MS analysis revealed that *Cw*TPS8 could convert FPP into multiple sesquiterpene products, with β-caryophyllene (1) and α-humulene (2) being the two primary products (**Figure 3A**). Kinetic profiles of *Cw*TPS8 for FPP are as follows: 28.17 µM (*K*_m_ of α-humulene), 27.99 µM (*K*_m_ of β-caryophyllene), 1.038 µM/min (*V*_max_ of α-humulene), 13.29 µM/min (*V*_max_ of β-caryophyllene), 0.002 s^-1^/µM (*K*_cat_/*K*_m_ of α-humulene), and 0.0254 s^-1^/µM (*K*_cat_/*K*_m_ of β-caryophyllene) (**Supplementary Figure S3**). In addition, *CwTPS8* was cloned into the eukaryotic expression vector pESC-Trp and transformed into the engineered yeast strain FY94, which we previously developed for the high-yield production of FPP (Hu et al., 2024). After fermentation and product extraction, the resulting compounds were analyzed by GC-MS. The results showed that *Cw*TPS8 is a multifunctional sesquiterpene synthase capable of producing five different sesquiterpene compounds (**Figure 3B**). Among these, β-caryophyllene (1) and α-humulene (2) are the primary products, and cryptomeridiol (5) is a minor product. Additionally, based on the retention index and mass spectrometry data, the other two products were identified as caryophyllenyl alcohol (3) and neointermedeol (4) (**Supplementary Figure S4**).

Collectively, our functional characterization demonstrates that *Cw*TPS8 is a multifunctional sesquiterpene synthase capable of catalyzing diverse cyclization reactions from the substrate FPP. Primarily, it catalyzes the 1,11-cyclization of FPP to generate β-caryophyllene (1), α-humulene (2) and caryophyllenyl alcohol (3). Additionally, the enzyme exhibits secondary 1,10-cyclization activity, producing neointermedeol (4) and cryptomeridiol (5).

### 3.3 Residues that affect the generation of products

**Figure 4:**
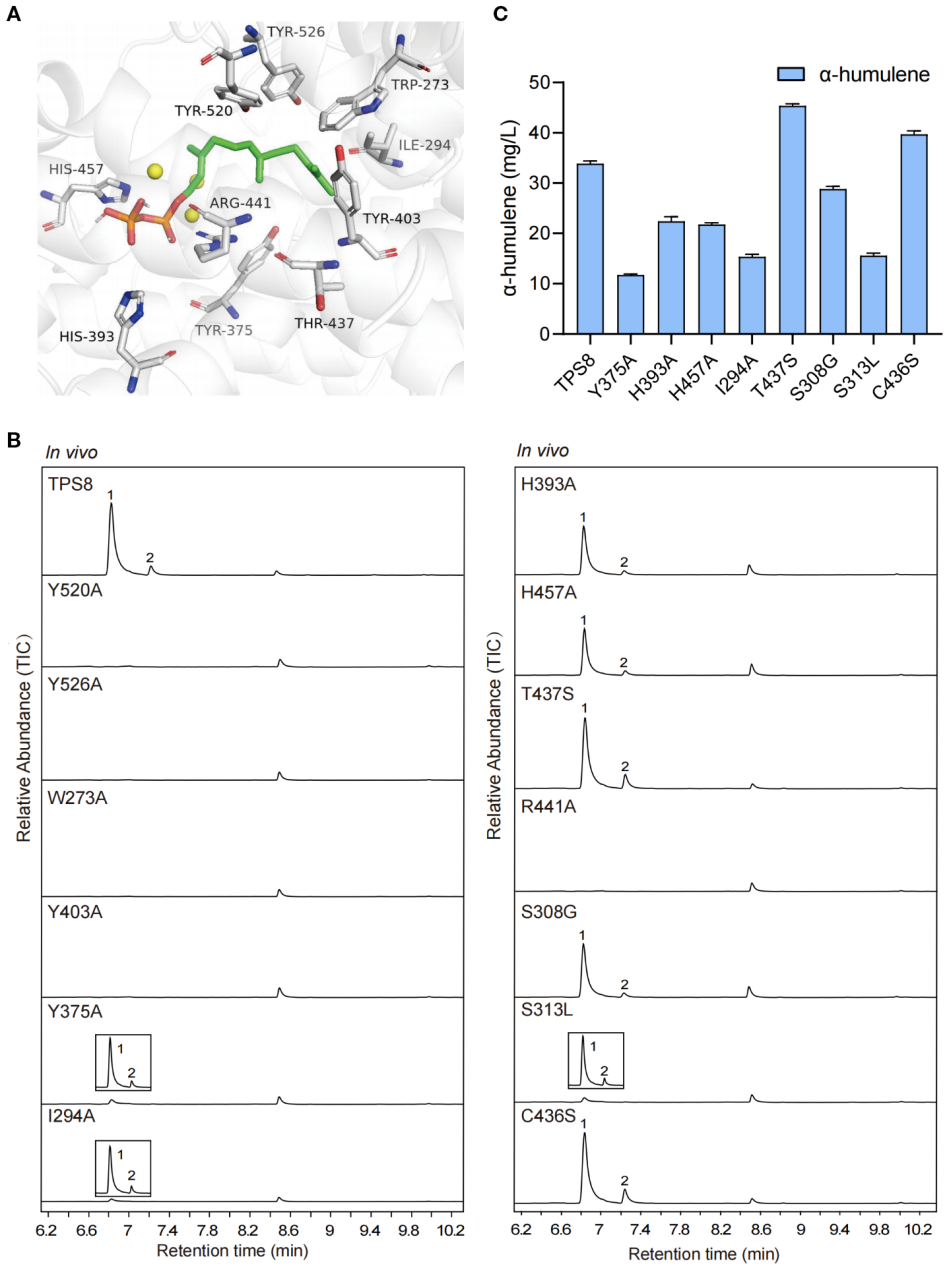
The molecular docking and site-directed mutagenesis research of *Cw*TPS8. **(A)** The molecular docking of *Cw*TPS8 and the substrate FPP. The labeled amino acids are key residues of the active pocket; **(B)** GC-MS analysis of products of the mutants. **(C)** The quantitative analysis of α-humulene in the mutants. Data are mean ± SD, *n* = 4.

Under the catalytic action of *Cw*TPS8, the pyrophosphate group of the substrate farnesyl pyrophosphate (FPP) undergoes cleavage to generate a farnesyl carbocation, which subsequently undergoes 1,11-cyclization to form the (*E, E*)-humulyl cation. This intermediate is then converted to α-humulene through a deprotonation step, while the biosynthesis of β-caryophyllene requires carbocation rearrangement of the (*E, E*)-humulyl cation followed by deprotonation (Yang et al., 2022). Single amino acid substitutions at or near the active site may critically influence catalytic efficiency and product specificity during this cascade. To find out the key residues affect the generation of products, site-directed mutagenesis experiments targeting key residues in the active site of *Cw*TPS8 were conducted based on molecular docking analysis. (**Figure 4A**).

The cyclization of the farnesyl carbocation is primarily influenced by the architecture of the active site, which consists of both aromatic and aliphatic residues. Aromatic residues stabilize reaction intermediates through cation-π interactions and direct cation migration toward specific cyclization pathways (Christianson, 2017). The aliphatic residues near the active site have been implicated in the cyclization and isomerization steps of certain sesquiterpene synthases (Gonzalez et al., 2014; Ker et al., 2020). First, we investigated the roles of the aromatic amino acids surrounding the active site, including Trp273, Tyr375, Tyr403, Tyr520, and Tyr526. These aromatic amino acids were substituted with alanine. The results revealed that the Y375A mutant significantly reduced the yields of the primary products, β-caryophyllene and α-humulene, while the W273A, Y403A, Y520A, and Y526A mutants completely abolished CwTPS8 activity (**Figure 4B**; **Supplementary Figure S5**). This finding demonstrates the critical role of aromatic side chains in stabilizing carbocation intermediates through π-π stacking. Next, we examined the function of the aliphatic residue Ile294 in the active site. After being replaced with alanine, the I294A mutant exhibited a dramatic reduction in product formation, with only hence β-caryophyllene and α-humulene were detected (**Figure 4B**). This result suggests that the hydrophobic side chains of aliphatic residues near the carbon chain of FPP, such as Ile294, are significant for stabilizing substrate intermediates through hydrophobic interactions.

In addition, previous studies have reported that the basic residues near the pyrophosphate group of FPP can facilitate the removal of the pyrophosphate group, promote the formation of carbocation intermediates, and ultimately lead to the generation of the products (Starks et al., 1997; Lopez-Gallego et al., 2010). In this study, we investigated the roles of Arg441, His393, and His457, which are located near the pyrophosphate group of FPP. All of these residues were substituted with the neutral amino acid alanine. The results showed that the H393A and H457A mutations exhibited a significant reduction in *Cw*TPS8 activity, while the R441A mutation directly inactivated *Cw*TPS8 (**Figure 4B**). These findings indicate that the positively charged residues surrounding the pyrophosphate group of FPP, such as Arg441, His393, and His457, play a crucial role in the activity of the sesquiterpene synthases.

Furthermore, we performed sequence alignments of *CwTPS8* with other α-humulene synthases and β-caryophyllene synthases. First, we identified a significant difference at residue 437 when comparing it to humulene synthases. In typical humulene synthases, this residue is a conserved serine, whereas in *CwTPS8*, it is a threonine (Thr437) (**Supplementary Figure S6A**). In light of this finding, we substituted Thr437 with serine. Notably, the resulting mutant, T437S, exhibited an increased production of α-humulene (37%) and a decreased production of another primary product, β-caryophyllene (**Figures 4B, C**; **Supplementary Figure S5**). This result may be attributed to the substitution of serine, which reduces steric hindrance by eliminating a methyl group. This alteration enhances hydrogen bonding between the serine hydroxyl group and the adjacent arginine residue. The strengthened hydrogen-bond network increases the local electropositivity of the guanidinium group of arginine, thereby modulating the spatial orientation of the humulyl cation. This electrostatic steering promotes deprotonation, facilitating the formation of α-humulene over C2-C10 cyclization, which is essential for the production of β-caryophyllene. Consequently, this effectively directs the carbocation intermediate toward the α-humulene pathway. Additionally, by comparing with β-caryophyllene synthases, we identified three relatively conserved amino acids and constructed the mutants S308G, S313L, and C436S (**Supplementary Figure S6B**). The results indicated that the S308G and S313L mutations resulted in varying reductions in *Cw*TPS8 activity, while the C436S mutation led to an 18% increase in the production of α-humulene, with the yield of β-caryophyllene remaining essentially unchanged (**Figures 4B, C**; **Supplementary Figure S5**). The effect of the C436S mutation may be similar to that of the T437S mutation, potentially influencing the quenching of the humulyl cation by enhancing hydrogen bonding with the adjacent arginine residue, thereby leading to an increase in α-humulene production.

### 3.4 Cloning and bioinformatics analysis of candidate short-chain dehydrogenase gene

After obtaining the key sesquiterpene synthase *Cw*TPS8 involving in zerumbone biosynthetic pathway, we tried to screen the CYP450 enzyme that capable of converting humulene into 8-hydroxy-α-humulene in *C. wenyujin*. However, unfortunately, we failed to find this CYP450. Instead, we continued to investigate the short-chain dehydrogenase (SDR) that catalyzes the downstream synthesis of zerumbone.

**Figure 5:**
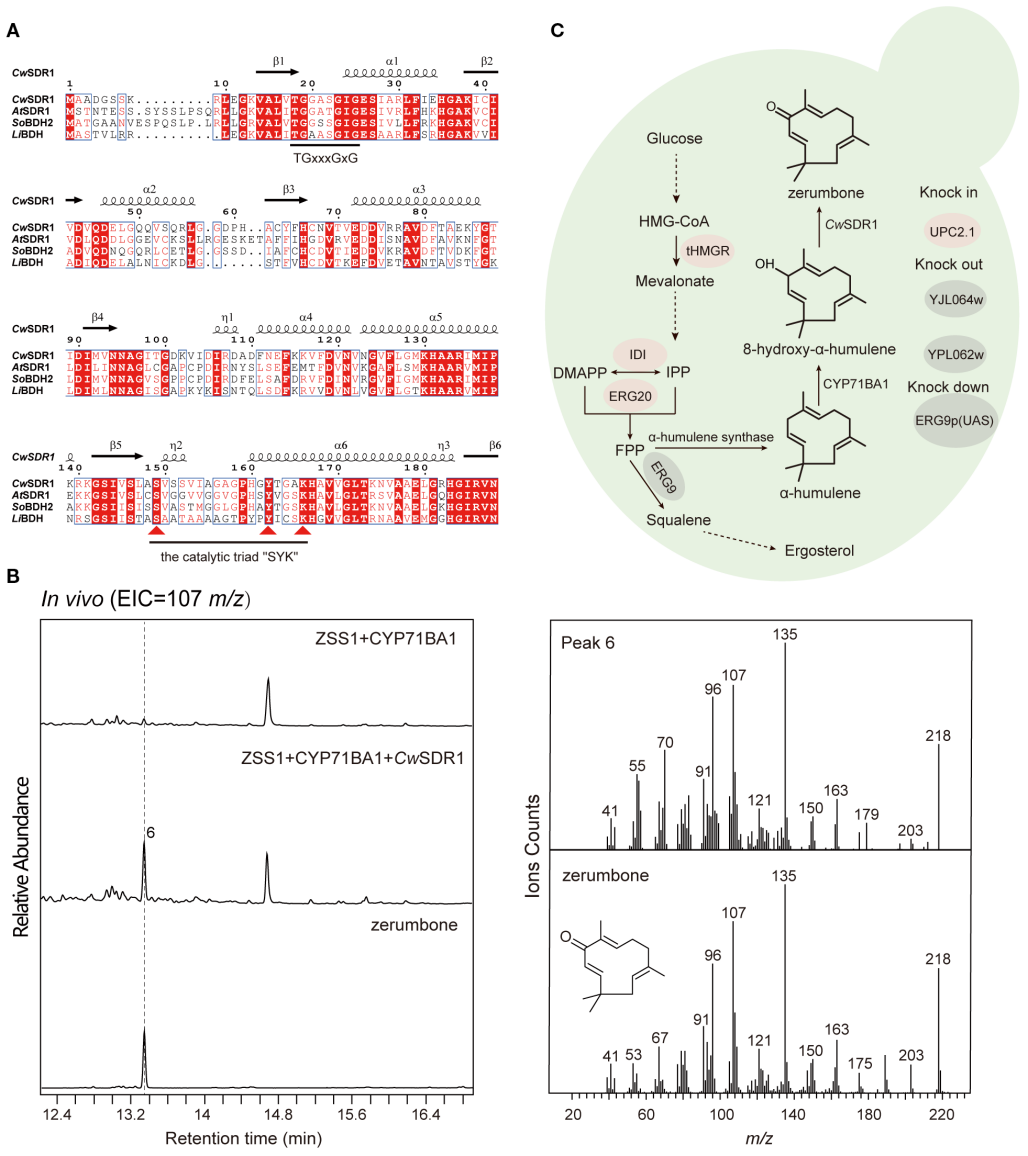
Sequence analysis and functional characterization of *CwSDR1* and the construction of a yeast strain for the *de novo* synthesis of zerumbone. **(A)** Sequence analysis of *CwSDR1*, containing the Rossmann fold, the conserved cofactor-binding motif “TGxxxGxG” and the catalytic triad “SYK” motif; **(B)** GC-MS analysis of the products of yeast strains expressing *Cw*SDR1; **(C)** Construction of the endogenous zerumbone biosynthetic pathway in yeast, with red highlighting overexpressed genes and gray indicating knockout/downregulated genes.

To identify the SDR responsible for zerumbone biosynthesis in *C. wenyujin*, we collected a subset of dehydrogenase genes from various plants, including *Lavandula angustifolia*, *Arabidopsis thaliana* and *Zingiber zerumbet*, which are involved in the generation of terpenoid dehydrogenation products (**Supplementary Table S3**). Additionally, three candidate short-chain dehydrogenase genes were selected based on the functional annotation of the transcriptomic data. Subsequently, we conducted a phylogenetic analysis of these candidate genes alongside the previously mentioned plant-derived terpenoid SDRs. The results indicated that one of the candidate genes, *CwSDR1*, clustered into a clade with the reference SDRs (**Supplementary Figure S7**), suggesting conserved catalytic roles in terpenoid metabolism. Sequence analysis revealed that *CwSDR1* is classified as an extended SDR, containing the characteristic Rossmann fold, the conserved cofactor-binding motif “TGxxxGxG” and the catalytic triad “SYK” motif (**Figure 5A**) (Zhang et al., 2023b).

### 3.5 *Cw*SDR1 catalyzes the conversion of 8-hydroxy-α-humulene into zerumbone

The gene *CwSDR1* was cloned into the eukaryotic expression vector pESC-Ura and co-transformed with *CYP71BA1*, which produces 8-hydroxy-α-humulene, into the engineered yeast strain with high-yield α-humulene, FY94-1 (**Supplementary Table S2**). GC-MS analysis revealed that 8-hydroxy-α-humulene was undetectable in the fermentation products of the control strain harboring *CYP71BA1*. Instead, a trace amount of zerumbone was observed, likely due to the spontaneous conversion of the unstable allylic secondary alcohol group in 8-hydroxy-α-humulene under the mildly acidic intracellular environment of yeast. In contrast, a pronounced peak corresponding to zerumbone was detected in the engineered strain expressing *CwSDR1* (**Figure 5B**). Quantitative analysis revealed a 357% increase in zerumbone production compared to the control strain. This result suggested that *CwS*DR1 is a short-chain dehydrogenase/reductase (SDR) that catalyzes the dehydrogenation of 8-hydroxy-α-humulene to zerumbone (6). Additionally, the strain FY94–3 that capable of *de novo* biosynthesis of the anticancer compound zerumbone was constructed (**Figure 5C**). However, the yield of zerumbone reached only 0.50 μg/L (1.88 μg/g DCW), with a specific productivity of 0.03 μg/g DCW/h. This low yield may be attributed to the limited catalytic efficiency of the natural CYP450 oxidase, CYP71BA1.

## 4 Discussion

This study successfully cloned and functionally characterized two pivotal terpenoid biosynthetic genes from *C. wenyujin*: the multifunctional sesquiterpene synthase *Cw*TPS8 and short-chain dehydrogenase/reductase (SDR) *Cw*SDR1. *In vitro*, *Cw*TPS8 catalyzes the conversion of FPP into β-caryophyllene (1) and α-humulene (2), which is a precursor of zerumbone. In *Saccharomyces cerevisiae*, *Cw*TPS8 not only produced these primary products but also generated cryptomeridiol (5) and two accessory sesquiterpenoids: caryophyllenyl alcohol (3) and neointermedeol (4). Enzyme *Cw*SDR1 was demonstrated to dehydrogenate 8-hydroxy-α-humulene into zerumbone (6). Mechanistic investigation of enzyme *Cw*TPS8 through molecular docking and site-directed mutagenesis identified critical amino acid residues that govern catalytic activity. The change of its spatial conformation will directly inactivate the enzyme or greatly reduce the product yield.

While β-caryophyllene and α-humulene are the primary products of *Cw*TPS8, the suboptimal yield of α-humulene restricts its utility in constructing high-titer zerumbone-producing microbial chassis. To address this bottleneck, future efforts will focus on enzyme engineering of *Cw*TPS8 through molecular dynamics (MD)-guided rational design and evolutionary divergence analysis, combined with site-directed mutagenesis to refine catalytic specificity. Concurrently, synthetic biology strategies—including the integration of strong constitutive promoters and amplification of plasmid copy number—will be implemented to enhance the efficiency of α-humulene production.

The suboptimal yield of zerumbone catalyzed by the enzyme *Cw*SDR1 may result from several bottlenecks: (1) insufficient activity of the P450 hydroxylase, which limits the supply of precursors; (2) inadequate NADPH regeneration capacity, constraining redox cofactor availability; and (3) the intrinsic catalytic inefficiency of *Cw*SDR1. To address these limitations, future research could employ QM/MM simulations to elucidate the mechanistic details of hydroxylation and oxidative dehydrogenation reactions. These insights would inform substrate channel engineering and the identification of rate-limiting residues for targeted mutagenesis. Concurrent strategies include (1) optimizing cofactor utilization through enhanced NADPH recycling via overexpression of NAD+/NADH kinases and NADP+ pyruvate dehydrogenase complexes; (2) enzyme engineering via high-throughput screening of evolved CYP450 libraries to identify hyperactive variants for the hydroxylation step; and (3) metabolic rewiring through dynamic regulation of the pentose phosphate pathway to rebalance NADPH flux.

This study comprehensively elucidates two pivotal stages in the biosynthetic pathway of zerumbone: the formation of the sesquiterpene scaffold and terminal functionalization. Furthermore, *Cw*TPS8 demonstrated catalytic promiscuity in both *in vitro* and *in vivo* systems, thereby expanding the enzyme repertoire for the diversification of sesquiterpenoids. These findings establish a theoretical framework for engineering high-yield terpenoid-producing *Saccharomyces cerevisiae* cell factories and optimizing the efficiency of zerumbone bioproduction.

## Data Availability

The datasets presented in this study can be found in online repositories. The names of the repository/repositories and accession number(s) can be found in the article/**Supplementary Material**.
