# Supplementary material for: Characterization of a sesquiterpene synthase and a short-chain dehydrogenase in zerumbone biosynthesis and the applications in engineered Saccharomyces cerevisiae
Source: Front Plant Sci. 2025 Oct 3;16:1635141. doi: 10.3389/fpls.2025.1635141 (PMC12532338; doi:10.3389/fpls.2025.1635141)
Supplement: Supplementary file 1 [file DataSheet1.docx]

Supplementary Material

## Supplementary Table 1. Primers used in this study.

| **Primer name** | **Sequence (5ʹ**-**3ʹ)** |
| --- | --- |
| **Primers used for cloning** | |
| TPS8-F | CTGATGATGGCCCAGCTTGT |
| TPS8-R | GCATTGCCCTGATAAGACATACTCT |
| SDR1-F | ATGGCAGCCGACGGTTCCT |
| SDR1-R | TTATTCGAACACTTGGAGTGTATGG |
| **Primers used for DNA assembly by seamless cloning assembly** | |
| TRP-TPS8-F | CGTCAAGGAGAAAAAACCCCGCTGATGATGGCCCAGCTTGT |
| TRP-TPS8-R | TAGTGAGTCGTATTACGGATCGCATTGCCCTGATAAGACATACTCT |
| URA-SDR1-F | ATCCTTGTAATCCATCGATATTATTCGAACACTTGGAGTGTATGG |
| URA-SDR1-R | CTCACTAAAGGGCGGCCGCAATGGCAGCCGACGGTTCCTCTAA |
| LEU-AtCPR-F | CGTCAAGGAGAAAAAACCCCGATGACTTCTGCTTTGTATGCTTC |
| LEU-AtCPR-R | TAGTGAGTCGTATTACGGATCTCACCAGACATCTCTGAGGTATC |
| His-TPS8-F | GGAAGGATTTCAGAATTCGGAATGATGGCCCAGCTTGTTGATGG |
| His-TPS8-R | GTGGTGGTGGTGGTGGTGGGAAATAGGAAAGGATTCAACCAATATG |
| **Primers used for the detection of mutant proteins** | |
| T437S-F | ATCCTTAAATCTTGCAGTATAATTTCTA |
| T437S-R | CTGCAAGATTTAAGGATCTTGGGGAAAC |
| W273A-F | GGTGGAATGTTATTATGCGATAGTTGCGG |
| W273A-R | GCATAATAACATTCCACCACTCGATCACG |
| Y375A-F | AGTTCTATCAAGATCTGCTTTCCAAGAAG |
| Y375A-F | GCAGATCTTGATAGAACTTTTATTTCCTC |
| H393A-F | GCCATCACTCGAGGAAGCTCTATGTGTTT |
| H393A-R | GCTTCCTCGAGTGATGGCACATATCCTTC |
| Y403A-F | GTTGAAATCCACAGGTGCTCCTGTGGTTA |
| Y403A-R | GCACCTGTGGATTTCAACGAAACACATAG |
| R441A-F | TTGCACTATAATTTCTGCACTTATGGATG |
| R441A-R | GCAGAAATTATAGTGCAAGATTTAAGGAT |
| H457A-F | GGAGCAAGAGAGAGACGCTGTAGCTTCAA |
| H457A-R | GCGTCTCTCTCTTGCTCCAACTCGTGTGA |
| Y520A-F | AGCCATGGAAGACATAGCCAAGCACAATG |
| Y520A-R | GCTATGTCTTCCATGGCTCTTGAAAGGTT |
| Y526A-F | CAAGCACAATGATACTGCTACTAATTCCA |
| Y526A-R | GCAGTATCATTGTGCTTGTATATGTCTTC |
| I294A-F | AATTACTTCCAAGGCTGCTTCCCTCATGT |
| I294A-R | GCAGCCTTGGAAGTAATTAATCGTGCACG |
| S308G-F | CATCTATGATAACTACGGCACATTGGAA |
| S308G-R | CGTAGTTATCATAGATGTCATCCATAAT |
| S313L-F | CAGCACATTGGAAGAGCTTCGATTATTAA |
| S313L-R | AGCTCTTCCAATGTGCTGTAGTTATCATA |
| C436S-F | AAGATCCTTAAATCTTCCACTATAATTT |
| C436S-R | GAAGATTTAAGGATCTTGGGGAAACTAG |

## Supplementary Table 2. Strains and plasmids used in this study.

| Strain name | Genotype | Source |
| --- | --- | --- |
| ***Saccharomyces cerevisiae*** | | |
| CEN-PK2-1C | *MATa*, *ura3-52 leu2-3 112 trp1-298 his3-Δ1*; *MAL2-8C; SUC2* | Stored in the lab |
| FY94 | CEN.PK2-1C, *rox1Δ erg9-218-175Δ yjl064wΔ ypl062wΔ* *His-P_TDH3_-ERG20-T_TPI1_-P_ADH1_-IDI-T_PGI_-P_GPM1_-tHMG1-T_ADH1_-P_ENO2_-UPC2.1-T_CYC1_* | Stored in the lab |
| FY94-1 | FY94, pESC-Trp:: P*_GAL1_*-*ZSS1*- T*_CYC1_* | This study |
| FY94-2 | FY94-1, pESC-Leu:: P*_GAL1_*-*AtCPR*-T*_CYC1_*-P*_GAL10_*-*CYP71BA1*-T*_ADH1_* | This study |
| FY94-3 | FY94-2, pESC-Trp:: P*_GAL10_*-*CwSDR1*-T*_ADH1_* | This study |
| FY95 | FY94, *Leu, P_GAL1_-ERG10-T_TPI1_-P_GAL10_-ERG13-T_PGI_-P_GAL1_-tHMG1-T_FBA1_-P_GAL10_-ERG12-T_PDC1_-P_GAL1_-ERG8-T_RPS2_-P_GAL10_-ERG9-T_TDH1_* | Stored in the lab |
| ***Escherichia coli*** | | |
| Transtee(DE3) | pMAL-His::*CwTPS8* | This study |

## Supplementary **Table 3.** Genes used in the phylogenetic analysis and sequence alignment.

| Gene name | Species | Reference |
| --- | --- | --- |
| **Terpene synthase** | | |
| ZSS1 | *Zingiber zerumbet* | AB247331.1 |
| *Am*DG2 | *Aquilaria malaccensis* | WDY97414.1 |
| *Ac*HS1 | *Aquilaria crassna* | AMQ67165.1 |
| *Ac*HS2 | *Aquilaria crassna* | AMQ67166.1 |
| *Ac*HS3 | *Aquilaria crassna* | AMQ67167.1 |
| *At*TPS27 | *Arabidopsis thaliana* | AAO85539.1 |
| *Os*TPS3 | *Oryza sativa* | DQ872158.1 |
| *Za*TPS6 | *Zanthoxylum ailanthoides* | UJH94382.1 |
| *Zp*TPS1 | *Zanthoxylum piperitum* | BBD88588.1 |
| *Cf*GerA | *Chamaecyparis formosensis* | QBM78437.1 |
| *Tc*TPS8 | *Taiwania cryptomerioides* | QGN65614.1 |
| *Sa*SQS2 | *Santalum album* | AIV42940.1 |
| *Sa*BS | *Santalum album* | AIV42941.1 |
| *Sa*SS | *Santalum album* | AGV01243.1 |
| *Sa*SSy | *Santalum album* | ADO87000.1 |
| *Sa*SQS1 | *Santalum album* | AIV42939.1 |
| *Tw*NES | *Tripterygium wilfordii* | AQA26342.1 |
| *Cs*NES | *Camellia sinensis* | ARQ20729.1 |
| *HI*STS1 | *Humulus lupulus* | EU760350.1 |
| **Dehydrogenase** | | |
| *At*SDR1 | *Arabidopsis thaliana* | Q9C826.1 |
| *Na*SDR | *Novosphingobium aromaticivorans* | WP_011906790.1 |
| *Li*BDH | *Lavandula x intermedia* | K4N0V2.1 |
| *So*BDH1 | *Salvia officinalis* | A0A8F5SIS3.1 |
| *So*BDH2 | *Salvia officinalis* | A0A8F5XX49.1 |
| *Sr*BDH1 | *Salvia rosmarinus* | UBN09140.1 |
| *Sr*BDH2 | *Salvia rosmarinus* | 6ZYZ_A(PDB) |
| *Mp*IPDH | *Mentha x piperita* | Q5C9I9.19 |
| *Wv*BDH2 | *Wurfbainia villosa* | **/** |
| *Ps*SDR | *Pisum sativum* | AAF04253.1 |
| *Pp*SDR | *Podophyllum peltatum* | AF352734.1 |
| *Dl*SDR | *Digitalis lanata* | AAW31720.1 |
| SDRvv | *Vibrio vulnificus* | 3UCE_A(PDB) |
| MOD1 | *Arabidopsis thaliana* | NP_849940.1 |
| 3βHSD/D1 | *Arabidopsis thaliana* | AAY28502.1 |
| LOC_Os06g44270 | *Oryza sativa* | NP_001389849.1 |
| ZSD1 | *Zingiber zerumbet* | F1SWA0.1 |


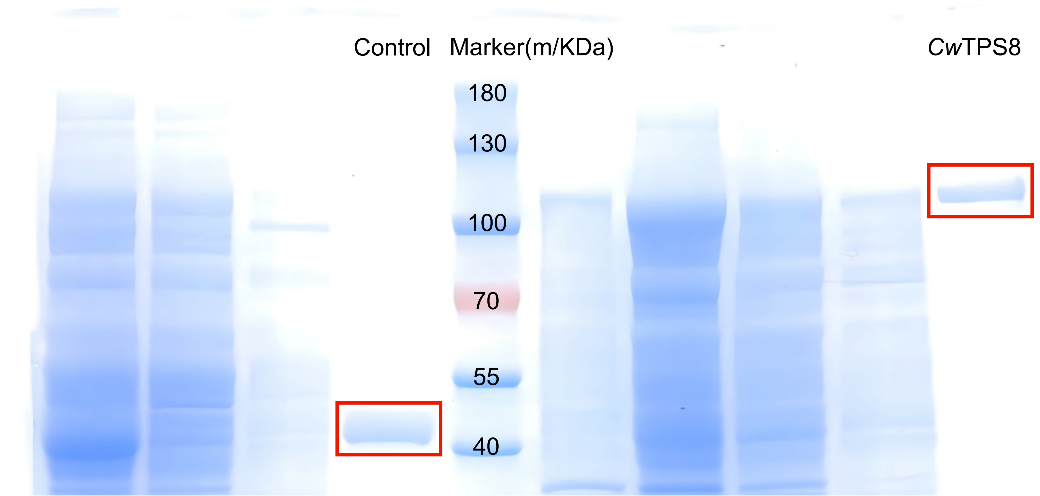


**Supplementary Figure 1.** The purified recombinant protein of *Cw*TPS8


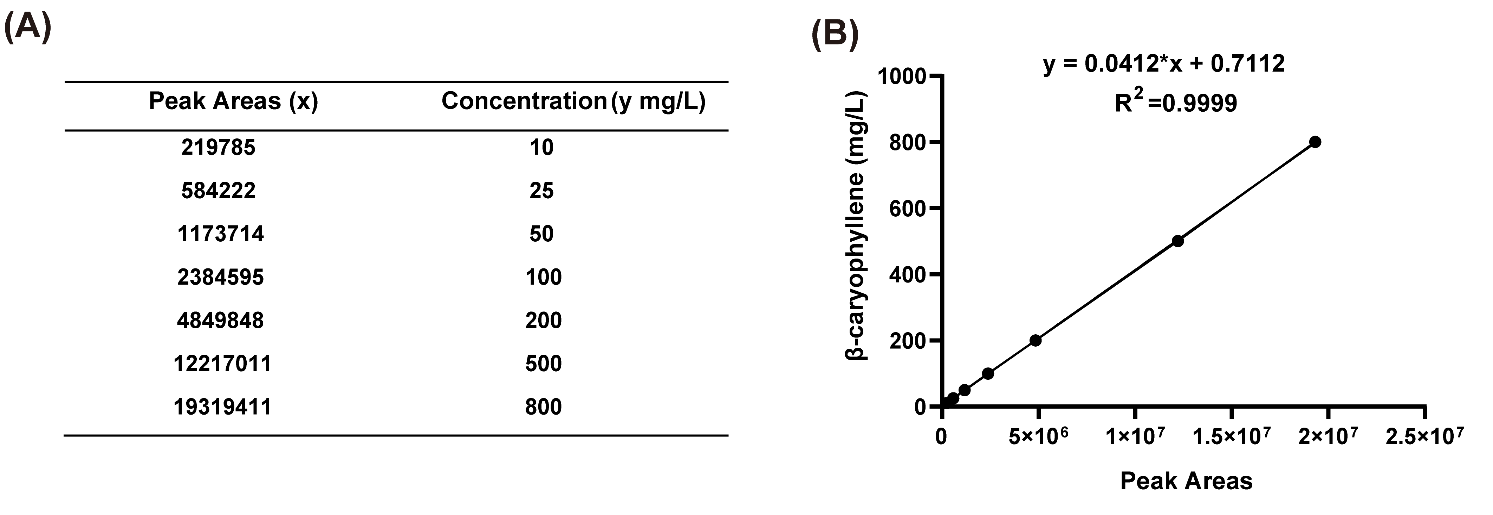


**Supplementary Figure 2.** Standard curve of β-caryophyllene

(A) Data used to calculate the β-caryophyllene standard curve. (B) Linear regression equation for β-caryophyllene.


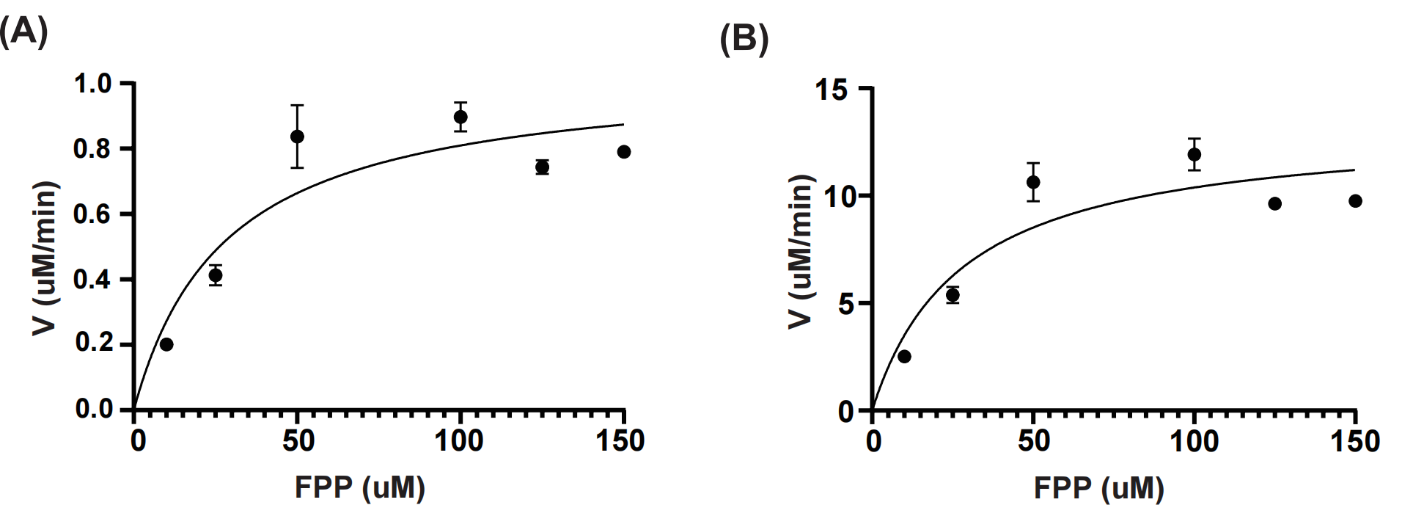


**Supplementary Figure 3.** Kinetic analysis of *Cw*TPS8

(A) Kinetic analysis of α-humulene in *Cw*TPS8; (B) Kinetic analysis of β-caryophyllene in *Cw*TPS8.

*K*_m_, *V*_max_, *K*_cat_ and *K*_cat_/*K*_m_ values were calculated using GraphPad Prism software. The data are presented as means ± SD of three replicates.


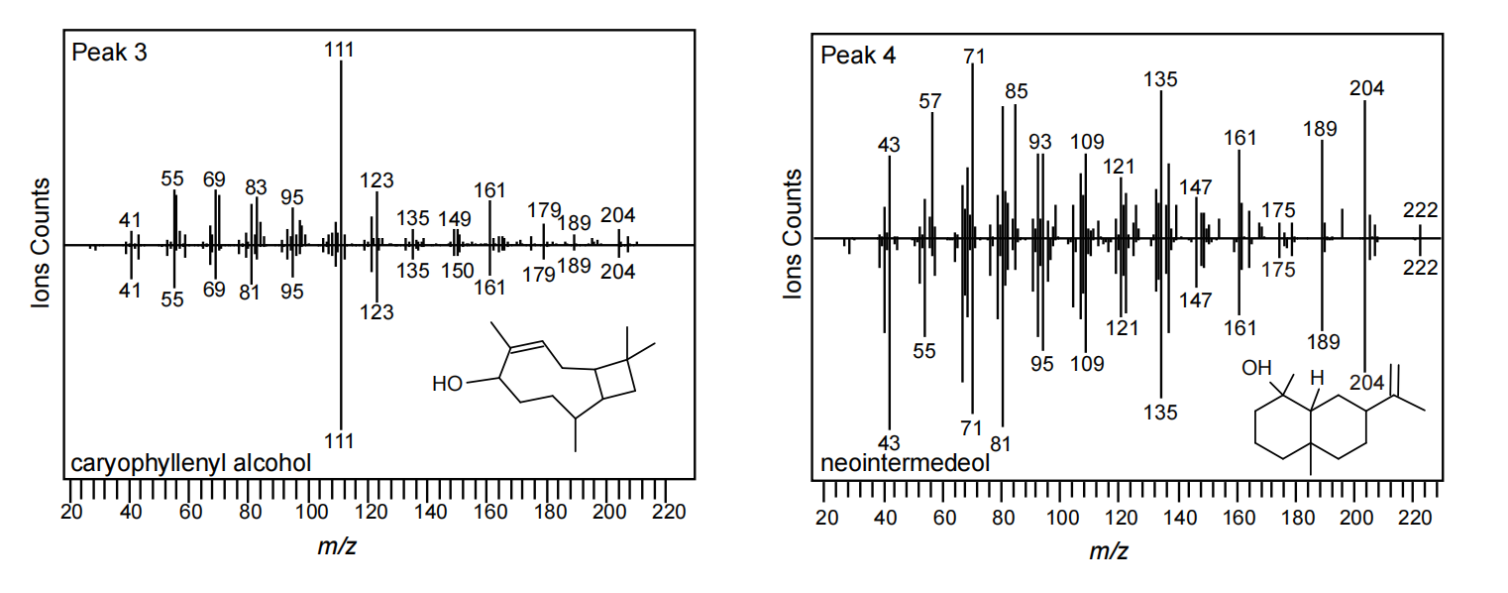


**Supplementary Figure 4.** Two other products of C*w*TPS8 analyzed using the NIST library


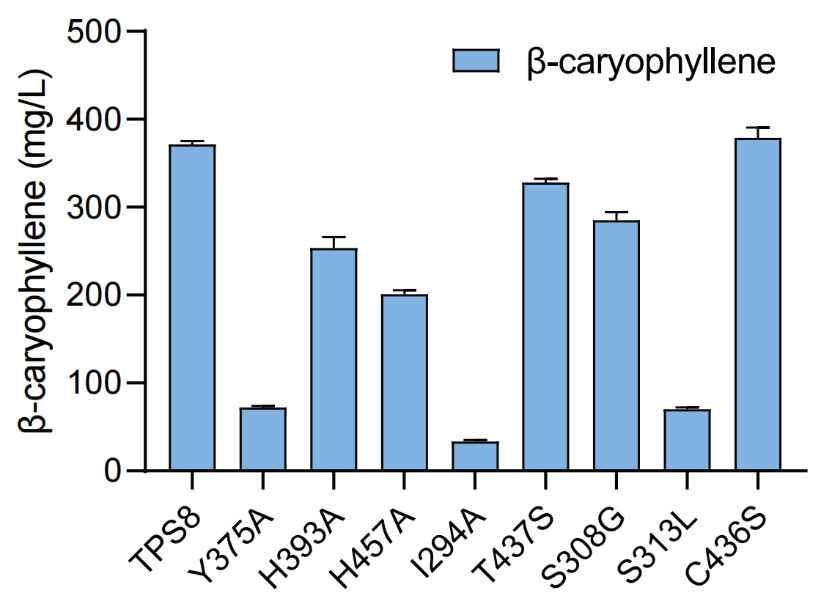


**Supplementary Figure 5.** The quantitative analysis of the β-caryophyllene of mutants formed from site-directed mutagenesis

Data are expressed as mean ±SD, *n*=4.


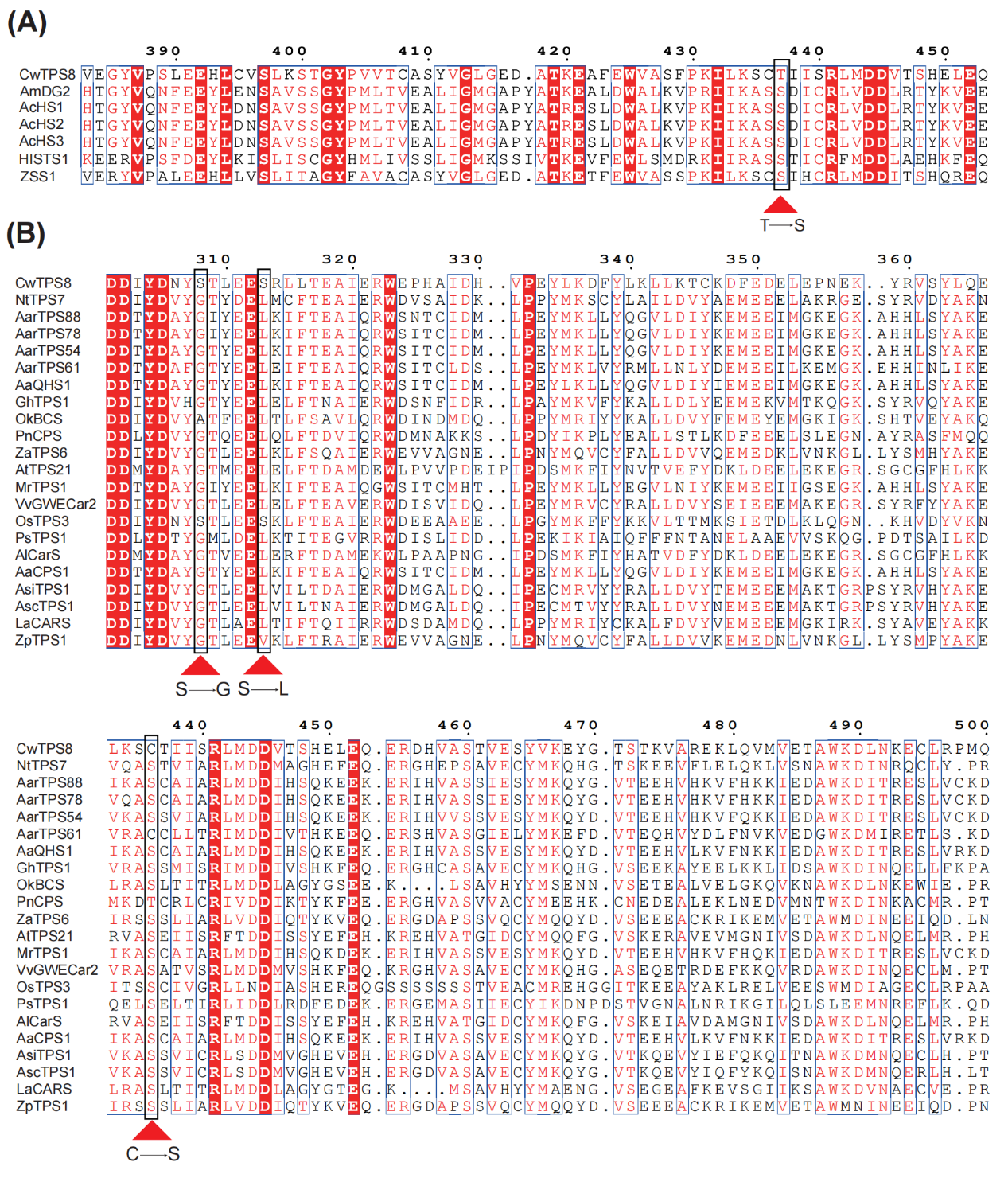


**Supplementary Figure 6.** Sequence alignments of *CwTPS8* with other α-humulene synthases and β-caryophyllene synthases

(A) The results revealed that position 437 in *CwTPS8* is threonine (T), whereas the corresponding position in other α-humulene synthase genes is serine (S)；

(B) Sequence alignment of *CwTPS8* with other β-caryophyllene synthase genes.


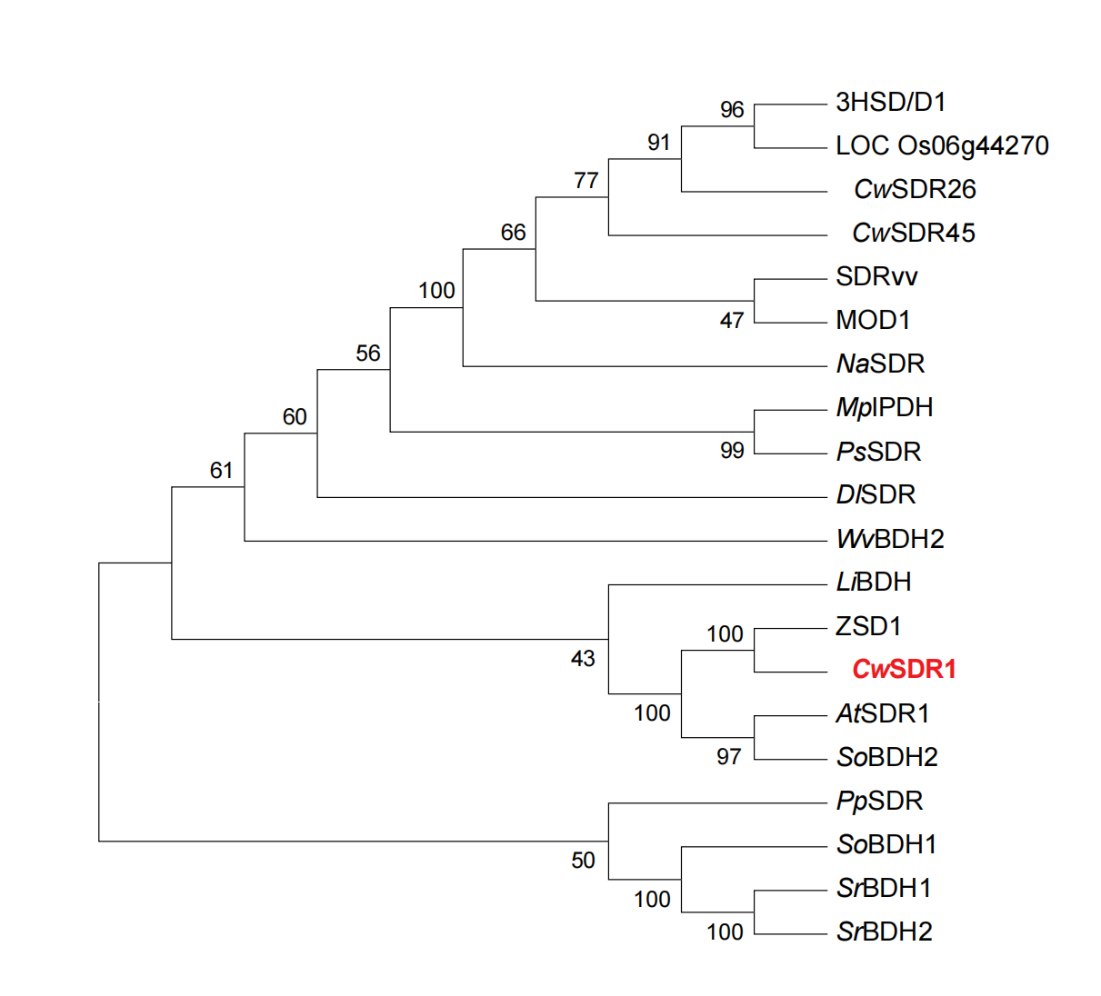


**Supplementary Figure 7.** The phylogenetic analysis of dehydrogenases
